# A set of ligation-independent in vitro translation vectors for eukaryotic protein production

**DOI:** 10.1186/1472-6750-8-32

**Published:** 2008-03-27

**Authors:** Viola Bardóczy, Viktória Géczi, Tatsuya Sawasaki, Yaeta Endo, Tamás Mészáros

**Affiliations:** 1 Budapest University of Technology and Economics, Department of Applied Biotechnology and Food Science, 1111 Budapest, Mûegyetem rkp. 3., Hungary; 2 Cell-Free Science and Technology Research Center, Ehime University, Bunkyo-cho 3-ban, Matsuyama 790-8577, Japan; 3 Pathobiochemistry Research Group of Hungarian Academy of Sciences and Semmelweis University, 1088 Budapest, Puskin u. 9., Hungary

## Abstract

**Background:**

The last decade has brought the renaissance of protein studies and accelerated the development of high-throughput methods in all aspects of proteomics. Presently, most protein synthesis systems exploit the capacity of living cells to translate proteins, but their application is limited by several factors. A more flexible alternative protein production method is the cell-free in vitro protein translation. Currently available in vitro translation systems are suitable for high-throughput robotic protein production, fulfilling the requirements of proteomics studies. Wheat germ extract based in vitro translation system is likely the most promising method, since numerous eukaryotic proteins can be cost-efficiently synthesized in their native folded form. Although currently available vectors for wheat embryo in vitro translation systems ensure high productivity, they do not meet the requirements of state-of-the-art proteomics. Target genes have to be inserted using restriction endonucleases and the plasmids do not encode cleavable affinity purification tags.

**Results:**

We designed four ligation independent cloning (LIC) vectors for wheat germ extract based in vitro protein translation. In these constructs, the RNA transcription is driven by T7 or SP6 phage polymerase and two TEV protease cleavable affinity tags can be added to aid protein purification. To evaluate our improved vectors, a plant mitogen activated protein kinase was cloned in all four constructs. Purification of this eukaryotic protein kinase demonstrated that all constructs functioned as intended: insertion of PCR fragment by LIC worked efficiently, affinity purification of translated proteins by GST-Sepharose or MagneHis particles resulted in high purity kinase, and the affinity tags could efficiently be removed under different reaction conditions. Furthermore, high in vitro kinase activity testified of proper folding of the purified protein.

**Conclusion:**

Four newly designed in vitro translation vectors have been constructed which allow fast and parallel cloning and protein purification, thus representing useful molecular tools for high-throughput production of eukaryotic proteins.

## Background

In the last decade, attention focused on functionality and structure of proteins. Accelerated proteomics studies demand high-throughput protein production methods to ensure availability of proteins of interest. Presently, overexpression in *E. coli *cells is the most preferred protein production method. Though this system has been well optimized and is suitable for the simultaneous generation of a panel of proteins, its application is often limited by the insolubility of synthesized eukaryotic proteins [1]. Although different *E. coli *strains and various protein and peptide fusion partners have been developed to increase the solubility of heterologous proteins, these methods are not universal and have to be optimized individually for efficient protein production [2].

Recently, in vitro protein translation has emerged as an alternative to cell-based protein synthesis methods. The robustness of the translation apparatus is known since the fifties, and latest technical improvements made to cell-free translation resulted in protein production methods that approach the efficiency of cell-based systems [3]. Various sources of translation machinery can be used for cell-free in vitro translation systems, but -due to its low cost and capacity for synthesizing properly folded, high molecular weight eukaryotic proteins- wheat germ derived protein extract presently seems the most promising choice [4].

Unlike prokaryotic mRNA, eukaryotic mRNA has to be extensively modified to be an effective translation template. The 5'-cap is essential to translation initiation and has to be introduced to in vitro transcribed mRNAs using RNA polymerase, which incorporates the three modified nucleotides (7-mG-5_-ppp-5_G). The efficiency of incorporation is low, and the excess of free modified nucleotides remaining in the mix dramatically decreases the productivity of translation. The 3'-end poly(A) tail of eukaryotic mRNAs also presents a technical difficulty during in vitro translation template preparation, as long polyA/T sequences of plasmids are unstable in host cells. To solve these problems, wheat germ in vitro translation vectors have been constructed with a special sequence replacing the cap. In the optimized vectors, the cap structure is substituted by either the tobacco mosaic virus translational enhancer Ω sequence with an additional GAA triplet at the 5'-end (GAA Ω) [5], or an artificial 73 nucleotides containing a leader sequence [6]. The same laboratory also examined the requirements for a poly(A) tail, and found that translation did not depend on the sequence but only on the length of 3'-UTR. An additional benefit of these plasmids is that the produced mRNAs were effective in vitro translation templates in a wider range of concentration than in vitro capped mRNAs.

Although the optimized vectors improved the productivity of in vitro translation, in order to build high-throughput protein synthesis systems, every step of the procedure must be accelerated, including the cloning of target genes and the purification of translated proteins. Ligation independent cloning (LIC) was developed to facilitate complex cloning and subcloning strategies [7], and have been applied by many laboratories since then. LIC overcomes important limitations of traditional cloning technologies, since any PCR product can be cloned into LIC compatible vectors without using restriction endonucleases and ligation. The LIC method takes advantage of the 3' exonuclease activity of T4 DNA polymerase to create complementary 12- to 15- nucleotide overhangs in the vector and PCR product. Upon transformation into *E. coli *cells, the host repair enzymes ligate at the vector-insert junction; thus, LIC produces high cloning efficiency with minimal non-recombinant background [8].

A serious bottleneck of high-throughput protein production is the fast and high level purification of target proteins. Generally, the purification step is facilitated by addition of affinity tags to the N- or C- terminus of synthesized proteins. Although the affinity tags aid the purification, it might in many cases alter the in vivo function and structure of proteins; hence, it must be removed by site specific proteases. The Tobacco Etch Virus (TEV) protease is an ideal choice because it cleaves with high specificity at a seven-amino-acid recognition sequence [9]. Furthermore, it is active under a wide range of conditions, such as low temperature and high ionic concentration, and is only mildly sensitive to many protease inhibitors which are used to prevent protein degradation by host proteases.

We have improved two commercial vectors for wheat germ in vitro protein translation to generate LIC plasmids incorporating a TEV cleavable affinity tag. The modified vectors encode a leader sequence consisting of either a GST or a His affinity purification tag, followed by a TEV protease recognition site. Experiments with the modified vectors showed that they functioned effectively in all aspects, including cloning, translation, purification and cleavage. Furthermore, we demonstrated that a protein kinase purified from a wheat germ in vitro translation reaction possessed higher in vitro kinase activity than the same kinase produced by overexpression in *E. coli*. These features make the modified vectors suitable for high-throughput production of properly folded eukaryotic proteins.

## Results and Discussion

### Construction and characterization of LIC vectors

**Figure 1:**
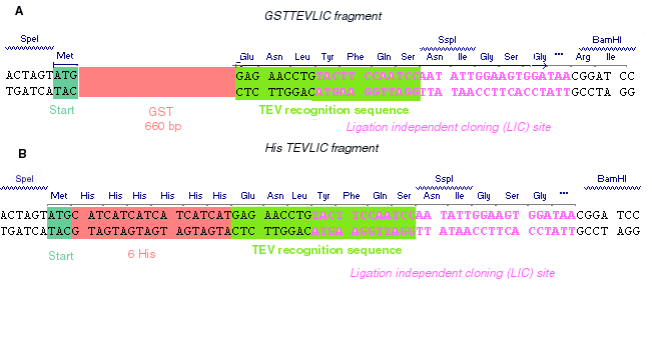
Nucleotide sequence of inserted cassettes.

mRNA templates of commercial in vitro translation systems are produced by either T7 or SP6 bacteriophage RNA polymerases, therefore we modified the pEU3N-II and pEU01 vectors which harbor T7 and SP6 promoters, respectively. The LIC vectors with cleavable affinity tags were created by inserting an oligonucleotide cassette or PCR-generated fragment into the multicloning site of host vectors. Sequencing of the constructed pEU3-NII-HLIC, pEU3-NII-GLIC, pEU-E01-HLIC, pEU-E01-GLIC vectors showed that the DNA fragments had been correctly introduced. Inserts in HLIC vectors encode an amino acid sequence consisting of an N-terminal methionine followed by a six-histidine affinity tag and the ENLYFQS TEV recognition site. The PCR fragment introduced into GLIC constructs possesses the same components except the six histidines are replaced by Glutathione-S-Transferase protein (Figure 1). The extensively used Gateway system shows limitations for protein production since the fusion partners and amino acids encoded by the recombination site cannot be removed without inserting protease specific motif coding nucleotides downstream of the recombination site, and non-native amino acids can interfere with the structure and functionality of purified proteins [10]. To obviate this drawback, an *SspI *restriction endonuclease site involving LIC was inserted in our vector constructs. This rational design places the TEV protease site in proximity of the native protein and allows removal of the affinity tag, leaving only three extra amino acid residues in N-terminal [11].

The created vectors were tested by generating constructs encoding a plant mitogen activated protein kinase, *At*MPK6. The vectors were cleaved in the middle of the LIC site by *SspI *digestion, and the linearized plamids were incubated with T4 DNA polymerase in presence of dGTP. Due to the 3'-5' exonuclease activity of T4 DNA polymerase, this protocol generated 15-base long single-stranded overhangs. To insert the target gene, the *At*MPK6 specific primers were designed with a 5'-end extension complementary to the LIC site, and the PCR products were treated with T4 DNA polymerase in presence of dCTP. The generated complementary overhangs allowed introduction of the target gene into the vectors treated with T4 DNA polymerase using a simple annealing step, without use of any other enzyme. The reaction mixture was directly transformed into competent cells, and colony PCR analysis showed than more than 90% of tested colonies carried the target gene, proving the LIC procedure to be very effective.

### *In vitro *translation of a mitogen activated protein kinase

**Figure 2:**
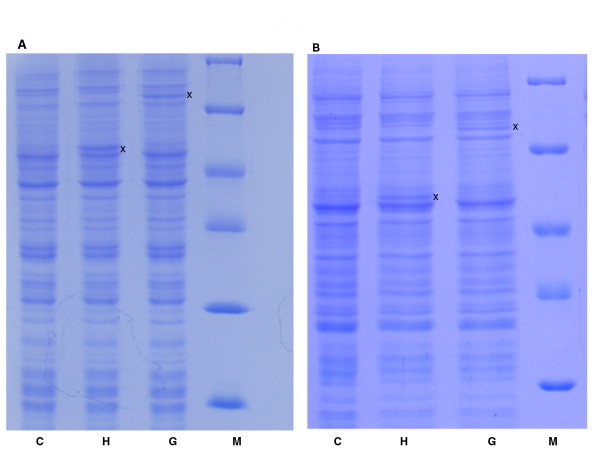
**SDS-PAGE analysis of in vitro translated proteins**. **A**. CEFC translation reactions with pEU3-NII backbone vector constructs: Molecular weight marker (M), wheat germ extract control (C), 1 μl out of 50 μl in vitro translation reaction mixture with pEU3-NII-HLIC (H) or pEU3-NII-GLIC (G) vector. **B**. Bilayer translation reactions with pEU-E01 backbone vector constructs: Molecular weight marker (M), wheat germ extract (C), 5 μl out of 225 μl in vitro translation reaction mixture with pEU-E01-HLIC (H) or pEU-E01-GLIC (G) vector. Proteins present in the translation mixtures were separated on 12% SDS-PAGE gel and detected by Coomassie Blue staining. Vector encoded-kinases are indicated by asterisks.

In order to test the protein synthesizing capacity of constructed vectors, two commercial wheat germ in vitro protein translation kits were used. These companies use different approaches to extend the lifetime and thus the productivity of in vitro translation reactions. The continuous supply of feeding solution is provided by diffusion through either a dialysis membrane [12] or simply the phase of different density solution [13], and they require T7 and SP6 RNA polymerase, respectively. The DNA templates were purified from *At*MPK6 comprising constructs with a commercial plasmid DNA isolation kit. The translation reactions were carried out according to protocols suggested by manufacturers. Polyacrylamide gel electrophoresis analysis of total protein samples demonstrated that all four vector encoded proteins with their expected size (Figure 2). The in vitro translated target proteins were detectable with Coomassie Blue staining, and the yields of different constructs and translation mixtures were comparable.

### Purification and TEV cleavage of translated proteins

**Figure 3:**
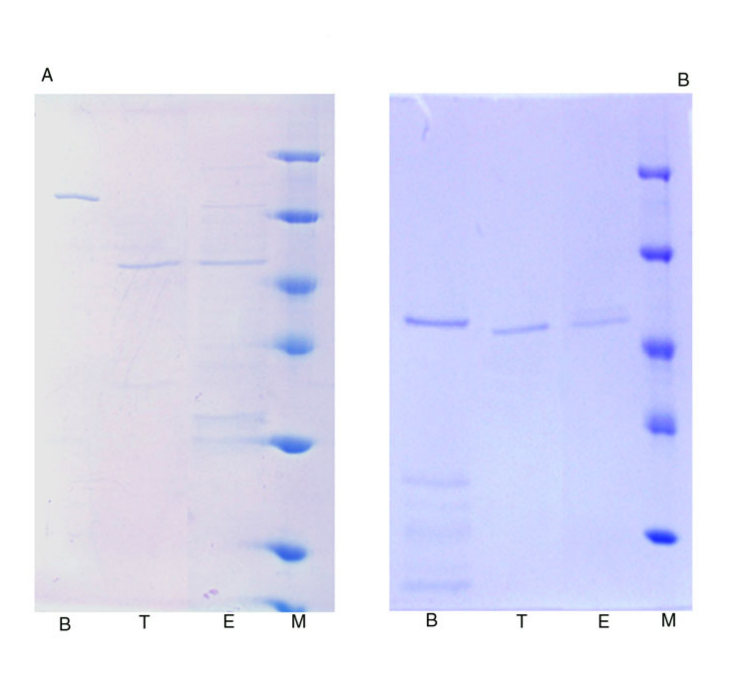
**Affinity purification and cleavage of translated proteins**. **A**. Purification and cleavage of GST*At*MPK6: Molecular weight marker (M), Glutathione-Sepharose coupled kinase (B), *At*MPK6 cleaved from the beads (T), *At*MPK6 cleaved following elution (E). **B**. Purification and cleavage of His*At*MPK6: Molecular weight marker (M), MagneHis particle coupled kinase (B), *At*MPK6 cleaved from the beads (T), *At*MPK6 cleaved following elution (E). Proteins were translated by CEFC and bilayer method, respectively. A quarter of the total amount of purified protein was separated on 12% SDS-PAGE gel and detected by Coomassie Blue staining.

To further verify the functionality of the created vectors, the synthesized proteins were affinity purified and cut by TEV protease. The GST- and His-tagged in vitro translation products were separated by batch incubation with Glutathione Sepharose and MagneHis particles, respectively. According to PAGE analysis, highly purified proteins were obtained within one hour of incubation for both affinity purification protocols (Figure 3). The TEV protease cleavage site was tested under different conditions. In the first case, the purified proteins were eluted with appropriate buffers and digested with His-tagged TEV protease. In the second case, the kinases were cut directly by TEV on the beads, without elution (Figure 3). The results demonstrated that the TEV protease worked effectively on both constructs under a wide range of conditions, since they were cleaved completely in different buffers, either coupled to beads or in solution.

### Kinase activity of the purified protein kinase

**Figure 4:**
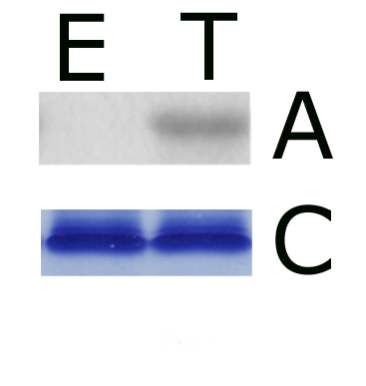
**In vitro kinase activity of translated versus overexpressed AtMPK6**. MBP phosphorylation activity of *E. coli *overexpressed (E) and translated (T) *At*MPK6 was detected by autoradiography (A). Coomassie Blue staining shows equal loading of MBP (C). *At*MPK6 was either produced in *E. coli *or translated by bilayer method, and 100 ng of purified kinase was used in kinase reactions.

Previously, it has been demonstrated that 207 out of 439 wheat germ in vitro translated plant protein kinases displayed autophosphorylation activity [14]. In order to compare the in vitro kinase activities of proteins produced in *E.coli *and in a cell-free system, the His tagged *At*MPK6 cassette was transferred from pEU3-NII-HLIC into pET11a expression vector. The kinase was isolated by metal chelate affinity chromatography either from *E. coli *or from the in vitro translation reaction mixture. Equal amounts of His-*At*MPK6 were used to determine the in vitro kinase activities of overexpressed and translated proteins using myelin basic protein (MBP) as a substrate. According to autoradiography results, phospholabeling of MBP was hardly detectable when bacterially overexpressed His-*At*MPK6 was tested, while the in vitro translated protein kinase displayed a clearly visible activity (Figure 4).

The high kinase activity of translated *At*MPK6 indicates proper folding of the kinase domain, although posttranslational modification(s) of the translated protein are also likely to be responsible for elevated kinase activity; indeed, proper phosphorylation of MAP kinases is essential to gain their full kinase activity and kinases present in wheat germ extract could perform these phosphorylations.

## Conclusion

We have constructed a set of four vectors to facilitate the cloning and purification steps of wheat germ extract protein in vitro translation systems. The presented vectors eliminate the traditional cloning steps and aid the purification of translated proteins by incorporation of a LIC site and a TEV cleavable affinity tag, respectively. Purification of a plant mitogen activated protein kinase demonstrated that the vectors functioned as intended. Furthermore, proper folding of the purified protein was indicated by high in vitro kinase activity. We have successfully used our vectors for purification of proteins with different sizes from various organisms, too (data not shown). In summary, these vectors allow fast and parallel cloning and protein purification, and hence represent useful molecular tools for in vitro translation of eukaryotic proteins.

## Methods

### Vector construction

The vectors pEU3-NII (Invitrotech) and pEU-E01 (CellFree Sciences) were used as backbones for plasmid construction. In order to remove the intrinsic *SspI *restriction endonuclease sites in pEU3-NII and pEU-E01, the vectors were mutated using Gene Tailor kit (Invitrogen) according to the provided manual with the 5'-ACTCTTCCTTTTTCAATGTTATGAAGCA-3' and 5'-TGAAAAAGGAAGAGTATGAGTATTCA-3';

5'-CTTCCTTTTTCAATGTTATTGAAGCATTTATCAGG-3' and 5'-CCTGATAAATGCTTCAATAACATTGAAAAAGGAAG-3' primers, respectively.

The mutated vectors were further manipulated to produce four different constructs.

#### pEU3-NII-GLIC, pEU-E01-GLIC

The LIC site combined GST fragment was generated by PCR. pGEX-2T (GE Healthcare) was used as template with primers 5'-ACTAGTATGTCCCCTATACTAGGTT-3'and 5'-GGATCCGTTATCCACTTCCAATATTGGATTGGAAGTACAGGTTCTCATCCGATTTTGGAGGATGGTC-3'. The PCR product was digested with *BamHI *and ligated into the *EcoRV-BamHI *digested pEU-3-NII *SspI- *vector, and transformed into DH10B competent cells. Ampicillin-resistant colonies were selected, and the purified plasmids were sequenced to confirm the PCR accuracy. The pEU-E01-GLIC plasmid was constructed by transferring GSTTEVLIC fragment from pEU3-NII-GLIC into pEU-E01*SspI- *vector. The fragment was obtained by *SpeI-BamHI *digestion and gel purification. The isolated fragment was inserted into pEU-E01*SspI- *vector treated with the same restrction endonucleases.

#### pEU3-NII-HLIC, pEU-E01-HLIC

The synthetic oligonucleotides

5'-TACTAGTATGCATCATCATCATCATCATGAGAACCTGTACTTCCAAT

CCAATATTGGAAGTGGATAACGGATCCA-3'and

5'-TGGATCCGTTATCCACTTCCAATATTGGATTGGAAGTACA

GGTTCTCATGATGATGATGATGATGCATACTAGTA-3'

encoding HisTEVLIC fragment were mixed, heated at 95°C for 30 sec, then incubated at 60°C for 3 minutes. The annealed, double stranded DNA was cut with *SpeI *and *BamHI*, and phenol-chloroform treatment of digested fragment was followed by ethanol precipitation. Finally, the fragment was ligated into *SpeI-BamHI *digested *SspI- *backbone vectors, and transformed into DH10B competent cells. The HisTEVLIC cassette comprising constructs were selected by colony PCR with primers specific for the backbone vectors:

pEUE01 forward: CGATTTAGGTGACACTATAGAACTC

pEU3-NII forward: CACTATAGGGTACACGGAATTCGC

pEU rev: TATAGGAAGGCCGGATAAGACG

### Ligation independent cloning

The following primers were designed to amplify *At*MPK6 and append the sequences required for LIC:

5'-TACTTCCAATCCAATGCA*ATGGACGGTGGTTCAGGT*-3' and

5'-TTATCCACTTCCAATG*TGTTTGAACGATCTGCAGTCA*-3' (gene specific sequences are indicated in italic). A vector construct comprising C-terminal HA-tagged *At*MPK6 [15] was used as template. The resultant PCR product was cleaned up by PEG precipitation protocol [16].

20 μg of pEU-LIC vectors were hydrolyzed by 100 U SspI (Fermentas) for 2 hours at 37°C separated on agarose gel, and purified with QUIAquick Gel Extraction Kit (Quiagen). The cohesive ends were generated by T4 DNA polymerase (Promega) treatment. Briefly, 1 μg linearized vector or PCR product was incubated for 10 minutes at 37°C with 1 U enzyme in presence of provided buffer and 1 mM dGTP or dCTP, respectively. The reaction was stopped by heat inactivation for 20 minutes at 75°C. 60 ng vector and 30 ng PCR fragment resulting from T4 DNA polymerase treatment were mixed and adjusted to 12.5 μl final volume with 5 mM EDTA concentration. The annealing mixture was incubated at RT for 20 minutes. The annealing mix was directly transformed into competent cells.

### Protein *in vitro *translation

2 μg of pEU-LIC*At*MPK6 constructs were used for mRNA synthesis, and the translation reactions were carried out according to the CEFC or bilayer protocol using commercial kits. In case of pEU3-NII backbone vector constructs, transcription and translation were done simultaneously at 23°C for 20 hours using RTS 100 WG CECF Kit (Roche). Constructs originating from pEU-E01 vector were transcribed by SP6 RNA polymerase, and half of the mRNA was added to the translation mixture of ENDEXT^® ^Wheat Germ Expression S Kit (CellFree Sciences). Transcription and translation were carried out as described in manual, and protein translation took place at 23°C for 20 hours.

### His affinity purification

The bilayer translation reaction mixture was incubated with 10 μl of MagneHis Protein Purification System (Promega) particles at 4°C for 30 minutes in 250 μl translation buffer completed with NaCl to 500 mM final concentration. After binding, the beads were washed five times with buffer containing 20 mM Tris, 10 mM imidazole, 500 mM NaCl, pH 7.5. The coupled His*At*MPK6 protein was removed from the beads either by elution with 20 mM Tris, 500 mM imidazole pH 7.5 buffer, or by cleavage with TEV protease treatment. TEV protease was purified essentially following a previously published protocol [17]. In order to directly digest kinase coupled to the beads, 2.5 μl beads were incubated with 1 μM TEV protease in washing buffer at 4°C, overnight. The eluted proteins were cleaved in elution buffer under the same condition.

### GST affinity purification

The translation reaction mixture was incubated with 10 μl Glutathione Sepharose 4B at 4°C for 1 hour in translation buffer, and the resin was washed four times with PBS to expel unspecific proteins. GST*At*MPK6 was eluted either by 20 minutes incubation in 50 mM Tris, 20 mM Reduced Glutathione pH 8.0 at 4°C, or cleaved on beads by TEV protease. TEV protease treatments were performed as described above.

### Production of AtMPK6 by bacterial overexpression

In order to compare the kinase activities of *E. coli *produced *At*MPK6 and in vitro translated *At*MPK6, the HisTEVLICMPK6 cassette was cloned into pET11a bacterial overexpression vector. Briefly, pEU-E01-HLICMPK6 was digested with *SpeI *and *BamHI*, and the resulting fragment was inserted into pET11a vector (Novagen) hydrolyzed with *NheI *and *BamHI *restriction endonucleases. The vector construct was transformed into *E. coli *BL21 (DE3) strain, and the cells were induced according to previously published protocol [18]. Briefly, the cells were grown at 37°C in 500 ml LB containing 100 μg/ml ampicillin until OD600 = 0.5, and incubated further 20 hours at 20°C with 0.1 mM IPTG (isopropyl-β-D-thiogalactopyranoside). Bacterial cells were collected by centrifugation at 4500 rpm for 20 minutes, resuspended in buffer containing 20 mM Tris, 500 mM NaCl, 10 mM imidazole, pH 8.0 and disrupted by sonication in the presence of 1 mM PMSF. Following centrifugation at 13000 rpm for 30 minutes the supernatant was mixed with 80 μl MagneHis particles for 30 minutes. Unspecific proteins were removed by washing the beads five times with lysis buffer, and the bound protein was eluted with 80 μl of 20 mM Tris, 500 mM imidazole pH 7.5.

### Kinase assay

100 ng in vitro translated or *E. coli *overexpressed *At*MPK6 protein was added to 20 μl kinase assay mixture (25 mM Tris, 1 mM EGTA, 1 mM DTT, 5 mM MgCl_2_, 1 mM MnCl_2_, 20 μM ATP, 1 mg/ml Myelin Basic Protein, 5 μCi [γ-^32^P]ATP, pH 7.5) [19]. The reaction mixture was incubated at room temperature for 30 minutes, then stopped by addition of 5 × Laemmli SDS buffer. The samples were separated on 15% SDS-PAGE gel and analysed by autoradiography.

## Authors' contributions

VB and VG implemented the cloning, expression and translation studies, and participated in the design of inserted cassettes. TS and YE designed the original vectors and helped the translation studies with essential advices. TM conceived of studies, carried out the kinase assays and drafted the manuscript. All authors read and approved the final manuscript.
